# Dr. Samuel S. Brame’s Case of Artificial Premature Labour

**Published:** 1842-07-30

**Authors:** Samuel S. Brame

**Affiliations:** Lowestoft


*Case of artificial premature Labor.* By SAMUEL S. BRAME, of Lowestoft.—Elizabeth Bristow, aged 28, has been married seven years, and pregnant six times. Having a slightly contracted pelvis, and generally very large children, she has never been able to give birth to a fœtus at the full period of utero-gestation, without submitting to the operation of embryotomy. Her first confinement took place in September, 1836, when she was delivered of a living child in the eighth month, no artificial means being used to bring on labour.

In the month of December, 1837, having gone her full time the operation of embryotomy was performed for the first time.

In October, 1838, I first attended her, when she gave birth to a seven months' child. On the 4th of September, 1839, embryotomy was the second time performed upon a nine months' fœtus. At that time I desired her, should she ever become pregnant again, to inform me of the fact at the end of the seventh month. In her fifth pregnancy she miscarried towards the conclusion of the sixth month, in November, 1840.

Not having forgotten my request made to her at the last operation, her mother called upon me, a short time since, to say that her daughter was again pregnant, and in her seventh month. Being childless, and naturally dreading to undergo an operation so distressing to the feelings of a mother, she earnestly requested me, if it were possible, to relieve her at once. I waited until I considered she was somewhat advanced in her eighth month ; and on Saturday, April 2, at 12 o'clock at noon, after administering a dose of castor oil, and subsequently an enema, I punctured the membranes, by introducing a pointed quill through the os uteri, using the index finger of the right hand as a director.

Half an hour after this operation about two pints of liquor amnii escaped, the patient experiencing at the same time slight pain in the abdomen. Irregular pains, preceded by shiverings, supervened ; on the following day, at noon, the os uteri was fully dilated. At six o'clock on the same day I administered a dose of the ergot of rye, which was repeated at seven o'clock, and at eight my patient was delivered of a living child, thirty-two hours after puncturing the membranes.

Both mother and child are doing well.—*Prov. Med. Journ.* April 23, 1842.

